# The Connotation of Variances in the Risk Predictors, Medications, Homocysteine, and Homocysteine Pathway Gene Polymorphisms with CVA/Stroke

**DOI:** 10.1055/s-0041-1722884

**Published:** 2021-02-09

**Authors:** Rizwan Masud, Aleem Ul Haq Khan, Aiman Farogh Anjum, Ghazala Jawwad, Zahid Azeem, Haider Zaigham Baqai, Shoaib Naiyar Hashmi

**Affiliations:** 1 Department of Physiology, CMH Kharian Medical College, Kharian, Pakistan; 2 Department of Biochemistry, CMH Kharian Medical College, Kharian, Pakistan; 3 Department of Physiology, Rawal Institute of Health Sciences, Islamabad, Pakistan; 4 Department of Biochemistry, AJ&K Medical College, Muzaffarabad, AJ&K, Pakistan; 5 Department of Medicine, Nafees Medical College, Islamabad, Pakistan; 6 Department of Pathology, CMH Kharian Medical College, Kharian, Pakistan

**Keywords:** cerebrovascular accidents, single nucleotide polymorphisms, stroke, transient ischemic attack, homocysteine

## Abstract

Cerebrovascular accidents (CVAs) are vascular multifactorial, multigenic ailments with intricate genetic, environmental risk influences. The present study aimed to establish affiliation of CVAs/stroke with blood parameters, differences in prescribed drugs consumption, and with differences in homocysteine pathway genes polymorphisms. The participants in study included controls *n* = 251, transient ischemic attack (TIA) patients *n* = 16, and stroke cases *n* = 122, respectively, (total participants, *n* = 389). The analyzed single nucleotide polymorphisms (SNPs) included C677T(rs1801133), A1298C(rs1801131) of methylene tetrahydrofolate reductase ( *MTHFR* ), A2756G(rs1805087) of methyl tetrahydrofolate homocysteine methyltransferase/methionine synthase ( *MS* ), and the A192G(rs662) of paraoxonase 1( *PON1* ) genes, all validated by tetra-primer allele refractory mutation system polymerase chain reaction (T-ARMS-PCR). The insertion deletion (I/D; rs4646994) polymorphism in angiotensin converting enzyme ( *ACE* ) gene was analyzed using routine PCR. All studied traits were scrutinized through analysis of variance (ANOVA), and later through regression analysis. Through ANOVA and multiple comparison, there was association of CVA with serum homocysteine, cholesterol, and with diastolic blood pressure readings. When data was subjected to regression, serum homocysteine and diastolic blood pressure (significant through ANOVA), as well as two additional traits, high-density lipoproteins (HDL), and rs1801133 MTHFR SNP sustained statistical significance and noteworthy odds in relation to CVA and stroke. The ailments affecting cerebral vasculature are mutifactorial, whereby genes, proteins, and environmental cues all exert cumulative effects enhancing CVA risk. The current study emphasizes that SNPs and variation in circulating biomarkers can be used for screening purposes and for reviewing their effects in stroke/CVA-linked risk progression.

## Introduction

Stroke/cerebrovascular accidents (CVAs) are common in both the developed nations as well as developing nations and pose a raised national and international socioeconomic burden. 1 Stroke represents weakness/loss of power in extremities, secondary to abnormal brain tissue perfusion, which in turn is subsequent to ischemic or hemorrhagic cerebral vessels. Ischemic stroke (following thrombosis or infarction) is much more common than the hemorrhagic cerebral disease, comprising more than four-fifths of the cases, with males affected more than females. 2 The prevalence of stroke numbers in millions and a great majority of affected patients suffer from lifelong debility, incapacity, and even fatal outcomes. Trivial stroke symptoms and transient ischemic attacks (TIAs) are additionally associated with higher risk of stroke as well as higher risk of inabilities. 3 Stroke-related death was around five million in 2006 and is expected to rise by nearly another three million within 10 years. 4 The slight decline in CVAs detected in developed nations is concomitant with augmented cases in developing nations, however, the occurrence of disease, surviving patients, and even fatalities are continuously showing a rising trend. 5 In Pakistan, there are four million stroke cases, with the rate of CVA/stroke being five to 10 times higher as compared with United Kingdom or United States. 6 7 The number of stroke afflicted persons, in Pakistan, was around 80 of every 0.1 million individuals in year 2010, and number will greatly increase in the coming years. 4 5

The risk factors associated with stroke/CVA include conventionally assigned ones such as old age, male sex, hypercholesterolemia, hypertension, smoking, cardiovascular diseases, coagulopathies, as well as advanced “newly recognized” ones such as genetic polymorphisms, mRNA variants, elevated homocysteine, fibrinogen levels, and CRP among others. 8 9 10 There is strong predisposition of male gender, increasing age, higher blood pressure, smoking and less optimal physical activity with advanced odds of stroke. 9 The genetic polymorphisms and hyperhomocysteinemia are newly identified stroke risk predictors, the polymorphisms being independently associated with stroke. 8 Methylene tetrahydrofolate reductase ( *MTHFR* ) gene is related to high homocysteine levels, vascular disease, and stroke. The two significant single nucleotide polymorphisms (SNPs) in *MTHFR* gene, C677T (rs1801133) and A1298C (rs1801131) are also strongly associated with risk of CVA/Stroke, with some studies exhibiting one SNP in favor of the other. 11 12 13 The rs1805087 SNP in methionine synthase ( *MS* ) gene although is related to thromboembolic phenomenon still it does not exhibit relation to stroke. 13 The stroke/CVA is high in patients with rs662 PON1 SNP and rs4646994 ACE SNP. 14 15

The medications used in hypertensive patients have disparate results in outcomes, with some drug regimens showing beneficial and others no role in stroke prevention. The use of angiotensin converting enzyme (ACE) inhibitors and β blockers either alone or in combination gives dissimilar results in end point occurrence of stroke and/or fatal outcomes. 16 17 Stroke in hypertensive patients developed despite using the forementioned antihypertensive medications, possible due to less optimal dosage or compliance to therapy. 18 Statin usage and low dose aspirin administration (more advantage in combination therapy) is additionally associated with lower risk of cerebral vascular disease outcomes. 10 19 Some studies report that prescription/use of diuretics, calcium channel blockers shows a declining trend in hypertension management, while others show an increased trend. Diuretics and calcium channel blockers (parallel to previously stated antihypertensive medication) reduced the risk of cardiovascular events and stroke in hypertensive patients. 18 The aim of the present study is to elucidate the role of serum-related traits, genetic polymorphisms and differences in antihypertensive medication as they relate to stroke/CVA.

## Subjects, Materials, and Methods

### Study Characteristics

The approval for the current study was obtained from Ethical Committee/Institutional Review Committee of the Combined Military Hospital Medical College, Kharian. All the participants in study provided written informed consent for the facts/information and use of samples. The samples of controls, TIA, and stroke cases were acquired from Rawalpindi district headquarter hospital (DHQ hospital), Rawalpindi General/Benazir Bhutto Hospital, and Islamabad Polyclinic Hospital, Islamabad, respectively. Patients with renal diseases, heart failure, coronary artery disease (CAD), and coagulopathies were excluded.

All participants in study, whether controls, TIA cases, and those with stroke were diagnosed hypertensives, on prescription (with patchy drug compliance history, more so in cases). The TIA and CVA cases presented in ER within 2 to 5 hours of onset of symptoms, major fraction of the patients presenting in the early morning hours. Patients with TIA develop symptoms for limited duration, are at an increased risk of developing complete stroke, and hence require distinct attention. The presenting features for the majority of cases included severe headache and inability to stand up or sit on getting up after sleep, and in some cases fall while getting up or walking. There was accompanying inability to talk or to move the limbs, with feeling of numbness, dizziness, and incoordination. The physical examination revealed altered consciousness, sensory, and motor deficits in one half of the body with loss of power and inability to move affected limbs on command. Imaging analysis “the computed tomography” (CT scanning) was performed for all the TIA and CVA cases to confirm the site, localization, type, and extent of CNS damage. The repeat CT scans were performed for stroke patients in which earlier scans were nonremarkable. In patients with suspected cardiac anomalies or suspected coagulation disorders, blood tests, electrocardiogram (ECG) or echocardiography was performed and patients with renal disease, coagulopathies, heart failure, and fibrillation were excluded from the study. The patients who exhibited marked improvement and recovery within a day of presentation and no findings on CT scan were labeled as TIA patients, and those who exhibited no physical recovery in their CNS symptoms and signs, and with positive findings on CT scan (for hemorrhagic or ischemic stroke) were labeled as cases for the current study. All participants, whether controls or cases, were hypertensive, mostly overweight, and exhibited sedentary life styles.

### Investigation of Clinical and Body Fluid/Biochemical Factors

Clinical assessment, (performed on presentation and during blood sampling), included general physical checkup, cardiac and neurological status valuation, blood pressure estimation (systolic SBP/diastolic DBP), temperature (°C), and electrocardiogram (ECG) to check for possible cardiac abnormalities as possible cause of stroke. Blood sampling was performed at daybreak, serum was extracted (stored at −20°C till analysis), and the factors checked included: lipid profiling analysis for the total cholesterol, total triglycerides, and high-density lipoprotein evaluation (mg/dL); homocysteine (µmol/L); and creatinine, blood sugar (mg/dL) estimation. Enzyme-linked immunosorbent analysis “ELISA” assay by the EIA kit (DRG International Inc., United States) provided foundation for homocysteine estimation, whereas remaining serum factors were analyzed by commercial AMP kits by “AMEDA Diagnostic” (GmbH, Germany). The participant data regarding drug intake was recorded by questionnaires, and the query about the usage and compliance for the class of drug being used, including the statins, aspirin, calcium channel antagonists, β antagonists, ACE antagonists, and diuretics was designated.

For SNP analysis, 5 to 7 mL of venous blood was collected in EDTA tubes, DNA extracted (from peripheral blood leukocytes), by routine method and stored at 4°C. The genomic sequences for studied SNPs: rs1801133 and rs1801131 ( *MTHFR* gene); rs662 ( *PON1* gene); rs1805087 ( *MTR* / *MS* ) gene; and 4s4646994 ( *ACE* gene) were queried through UCSC genome browser database at http://genome.ucsc.edu/cgi-bin/hgGateway and primers were inquired through http://cedar.genetics.soton.ac.uk . The primers were optimized for obtaining appropriate and adequately amplified bands. Tetra-primer allele-refractory mutation system-polymerase chain reaction ARMS-PCR was performed, using 25-µL master mix, for all the studied SNPs and thermal cycler used for sequence amplification was Biometra T1 (GmbH, Germany). A random group of samples was subjected to restriction fragment length polymorphism analysis for authentication of genotype as attained through tetra-primer ARMS-PCR analysis. The primer sequences used, the reaction mixture details, the steps of genotyping techniques have been stated elsewhere. 20 21

### Statistical Analysis

Prior to analysis, the levels of homocysteine were converted to log values, and these log values were used, as the original values were significantly askew (established through R “nortest”). Analysis of variance (ANOVA) test and multiple comparison testing “post hoc” was performed using IBM SPSS v25 (IBM Corp. release 2017). ANOVA and post hoc scrutiny were computed for age, gender, lipid profile, homocysteine, blood sugar, blood pressure, drugs administered, and the related SNPs in the study; significant value was assigned for *p* values < 0.05. For post hoc analysis, we used the “Tamhane test” as this test gives values for variables assuming unequal variance in the data. Succeeding the ANOVA investigation was the regression scrutiny of the data through R 4.0.2 statistics package (R Core Team, 2020, Vienna, Austria). The data was analyzed for statistical significance, odds ratio, and 95% confidence intervals. All the traits examined through the ANOVA were analyzed through the regression investigation. The significance was again given to *p* -value < 0.05. As is the case with data in tables, the plots and graphs in the figures were also obtained through use of both the statistical packages. Both tests were performed for added scrutinization and authentication of the data.

## Results

### Descriptive/Demographic Data

The participant demographic data and biochemical variables, comparing the three test groups, are summarized in Table 1 . The number of males in controls ( *n* = 251) was 198 (78.9%), in TIA patients ( *n* = 16) was 13 (81.2%), and in CVA/stroke cases ( *n* = 122) was 94 ((77%); the mean age (± SD) was 65.2 years (8.09), 64.19 years (4.43), and 65.28 years (7.9) in the three forementioned groups, respectively. The mean values (± SD) of total cholesterol and creatinine in the controls were 195.68 mg/dL (42.94) and 1.006 mg/dL (0.42); in the TIA patients 190.84 mg/dL (36.07) and 0.975 mg/dL (0.27); and in the stroke cases was 206.66 mg/dL (37.11) and 1.001 mg/dL (0.36), respectively. Fasting blood sugar levels, HDL, and triglyceride levels (± SD) in controls, TIA patients and CVA cases were 109.91 mg/dL (42.85), 115.84 mg/dL (26.79), 118.72 mg/dL (54.81); 53.36 mg/dL (15.72), 50.75 mg/dL (14.72), 56.03 mg/dL (21.15); and 137.06 mg/dL (85.14), 134.44 mg/dL (15.95), 152.84 mg/dL (69.09), respectively. The systolic blood pressure (± SD) reading was 135.76 mm Hg (17.37), 133.88 mm Hg (9.27), and 137.87 mm Hg (21.43), whereby the diastolic blood pressure reading was 77.58 mm Hg (10.94), 78.25 mm Hg (5.08), and 80.99 mm Hg (12.59), respectively in the three studied groups. Finally, the log homocysteine levels in the controls was 1.12 µmol/L (0.12), in TIA patients' levels for log homocysteine were 1.08 µmol/L (0.10), and in CVA/stroke cases levels were 1.15 µmol/L (0.14).

**Table 1:** Patients/participant characteristics

Characteristics	Control, *n* = 251	TIA cases, *n* = 16	Stroke, *n* = 122
Age (years)	65.20 (8.09)	64.19 (4.43)	65.28 (7.9)
Gender			
Male (Percent)	198 (78.9%)	13 (81.2%)	94 (77.0%)
Female (Percent)	53 (21.1%)	3 (18.8%)	28 (23.0%)
C.t (mg/dL)	195.68 (42.94)	190.84 (36.07)	206.66 (37.11)
Cr. (mg/dL)	1.006 (0.42)	0.975 (0.27)	1.001 (0.36)
FBS (mg/dL)	109.91 (42.85)	115.84 (26.79)	118.72 (54.81)
TGl (mg/dL)	137.06 (85.14)	134.44 (15.95)	152.84 (69.09)
HDL levels (mg/dL)	53.36 (15.72)	50.75 (14.72)	56.03 (21.15)
SBP (mm Hg)	135.76 (17.37)	133.88 (9.27)	137.87 (21.43)
DBP (mm Hg)	77.58 (10.94)	78.25 (5.08)	80.99 (12.59)
Hcy(log), µmol/L	1.12 (0.12)	1.08 (0.10)	1.15 (0.14)

Abbreviations: C.t, total cholesterol level; Cr, creatinine level; DBP, diastolic blood pressure; FBS, fasting blood sugar; HDL, high-density lipoproteins; SBP, systolic blood pressure; TGl, triglyceride levels. Hcy(log), homocysteine log transformed values.

Note: The continuous variables are presented as mean (standard deviation) and the categorial variables are presented as number (percentage).

### Analysis of Variance and Multiple Comparison

All the variables/factors were analyzed through ANOVA and post hoc analysis, latter equation utilizing Tamhane test sighting unequal variance of data. Homocysteine levels had overall significance *p* = 0.008, and there was post hoc analysis documented significance in control versus TIA patients at *p* = 0.038 and between controls and CVA/stroke cases at *p* = 0.042. Total cholesterol levels also achieved statistical significance with *p* = 0.04 and post hoc analysis was significant in control versus stroke cases with *p* = 0.035. Finally, the diastolic blood pressure maintained significance at *p* = 0.025 with subsequent measured significant difference in controls compared with stroke cases at *p* = 0.034. Neither the differential drug usage nor the differential genotypes in the controls, TIA patients, and cases could achieve statistical valuation through the ANOVA analysis. The ANOVA results/post hoc analysis results are tabulated as Table 2 .

**Table 2:** The relationship of studied traits with stroke, based on ANOVA analysis and post hoc multiple testing

Study parameter	*p* (ANOVA)	Post hoc significance (between groups)		
		Control: TIA	TIA: stroke	Control: stroke
Gender	0.886	0.994	0.973	0.971
Hcy(log), µmol/L	0.008 a	0.376	0.038 a	0.042 a
C.t (mg/dL)	0.04 a	0.942	0.310	0.035 a
Cr. (mg/dL)	0.947	0.965	0.956	1.000
FBS (mg/dL)	0.225	0.806	0.981	0.324
TGl (mg/dL)	0.182	0.972	0.046	0.163
HDL (mg/dL)	0.289	0.876	0.517	0.524
SBP (mm Hg)	0.509	0.851	0.477	0.722
DBP (mm Hg)	0.025 a	0.955	0.308	0.034 a
Statins	0.527	0.996	0.901	0.626
Aspirin	0.821	0.909	0.940	0.997
Beta blockers	0.240	0.946	0.996	0.243
Calcium channel blockers	0.398	0.999	0.962	0.456
Diuretics	0.158	0.897	0.999	0.168
*ACE* inhibitors	0.271	0.671	0.961	0.431
Age	0.768	0.801	0.646	0.957
Family history of CHD	0.247	0.602	0.920	0.458
rs1801133 *MTHFR*	0.064	0.687	0.996	0.098
rs1801131 *MTHFR*	0.141	0.996	0.877	0.178
rs1805087 MTR	0.231	0.369	0.807	0.406
rs662 PON1	0.265	0.415	0.837	0.455
rs4646994 ACEindel	0.194	0.842	0.996	0.261

Abbreviations: C.t, total cholesterol level; Cr, creatinine level; DBP, diastolic blood pressure; FBS, fasting blood sugar; HDL, high-density lipoproteins; SBP, systolic blood pressure; TGl, triglyceride levels; Hcy(log), homocysteine log transformed values.

Note: The *p* -value computed through ANOVA; multiple comparison performed with assumptions of nonuniform data variance.

a *p* < 0.05.

### Regression Analysis

Post ANOVA, regression analysis gave different results, the cholesterol levels lost significance whereas other three constants, that were not significant in ANOVA, gained regression analysis-mediated significant result. Two of the three coefficients, although achieved statistical significance, yet had nonsignificant odds ratio as well as nonsignificant 95% confidence intervals, explicitly, serum creatinine level *p* = 0.038, OR = 0.732, 95% CI = 0.546 to 0.983, and systolic blood pressure *p* = 0.030, OR = 0.992, 95% CI = 0.985 to 0.999. The robust association of homocysteine with stroke remained vigorous following regression scrutiny as well with *p* = 0.019, OR = 3.075, 95% CI = 1.203 to 7.859, respectively. The diastolic blood pressure also retained significance, *p* = 0.001, OR = 1.02, 95% CI = 1.008 to 1.033. The third coefficient, nonsignificant following ANOVA, yet significant through regression analysis was rs1801133 MTHFR SNP; the *p* -value = 0.043, OR = 1.189, and 95% CI = 1.010 to 1.406, respectively. The results of the regression analysis for all the studied variables in the study are charted in the Table 3 .

**Table 3:** The relationship of studied traits with stroke based on regression analysis (with associated odds ratio, confidence intervals)

Study parameter	*p* (Regression analysis)	OR	95% CI
Gender	0.972	0.996	0.788–1.258
Hcy (log) µmol/L	0.019 a	3.075	1.203–7.859
C.t (mg/dL)	0.470	1.001	0.998–1.004
Cr. (mg/dL)	0.038 a	0.732	0.546–0.983
FBS (mg/dL)	0.280	1.001	0.999–1.003
TGl (mg/dL)	0.147	1.001	1.000–1.002
HDL (mg/dL)	0.049 a	1.006	1.000–1.013
SBP (mm Hg)	0.030 a	0.992	0.985–0.999
DBP (mm Hg)	0.001 b	1.020	1.008–1.033
Statins	0.581	0.941	0.758–1.169
Aspirin	0.543	1.065	0.869–1.304
Beta blockers	0.485	0.928	0.751–1.146
Calcium channel blockers	0.638	1.049	0.859–1.281
Diuretics	0.262	1.120	0.919–1.365
*ACE* inhibitors	0.520	1.067	0.876–1.300
Age	0.574	1.004	0.991–1.017
Family history of CHD	0.633	0.954	0.788–1.156
rs1801133 *MTHFR*	0.043 a	1.189	1.010–1.406
rs1801131 *MTHFR*	0.078	1.170	0.982–1.393
rs1805087 *MTR*	0.348	0.928	0.794–1.085
rs662 *PON1*	0.347	1.071	0.928–1.234
rs4646994 *ACE* indel	0.236	0.913	0.786–1.061

Abbreviations: C.t, total cholesterol level; Cr, creatinine level; DBP, diastolic blood pressure; FBS, fasting blood sugar; HDL, high-density lipoproteins; SBP, systolic blood pressure; TGl, triglyceride levels; Hcy(log), homocysteine log transformed values.

Note: The *p* -values computed through regression analysis.

a *p* < 0.05–0.005.

b *p* < 0.005.

**Fig. 1:**
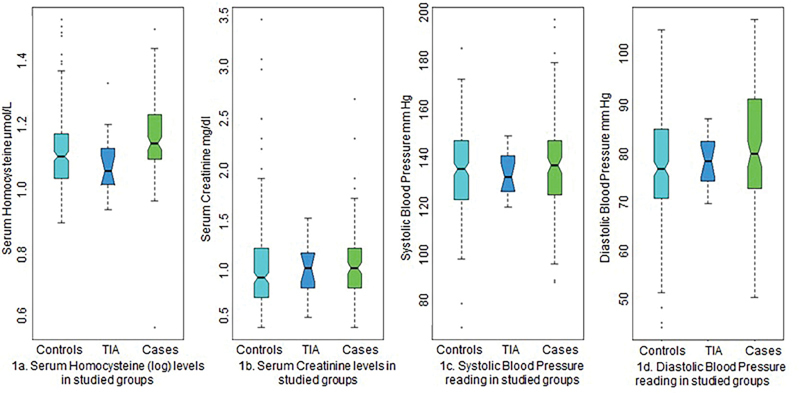
Graphic display of blood parameters and blood pressure readings in the three studied groups (controls, TIA, stroke cases) ( **A** ) serum homocysteine (log values) in the participants, ( **B** ) Serum creatinine levels in the participants, ( **C** ) systolic blood pressure (SBP) reading in participants, and ( **D** ) diastolic blood pressure (DBP) readings in participants, respectively.

**Fig. 2:**
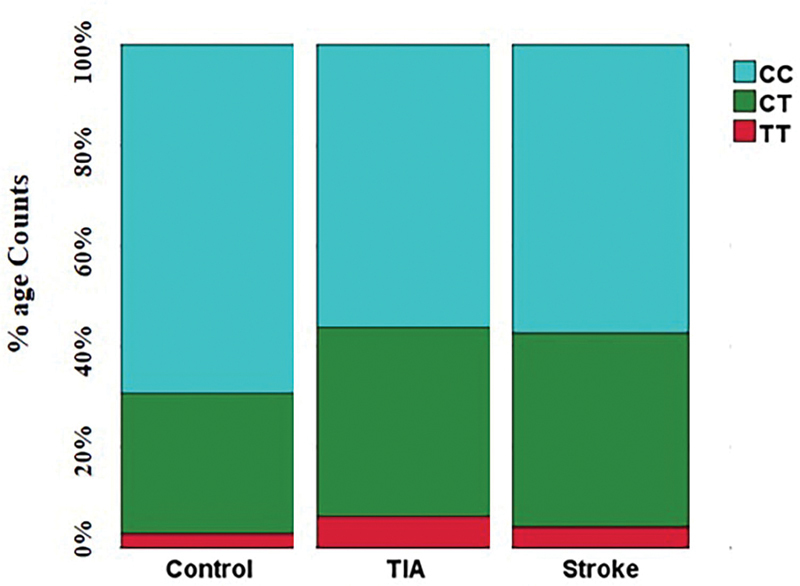
Graphic display (percentage frequency distribution) regarding allelic differences in the significant homocysteine pathway gene polymorphism; *MTHFR* rs1801133 (amongst the controls, TIA patients, and stroke cases).

The differences in the serum homocysteine (log) levels, serum creatinine levels, systolic and diastolic blood pressure readings amongst the controls, TIA cases, and stroke cases are plotted in Fig. 1 . The percentage frequency distribution of rs1801133 MTHFR SNP allelic differences in controls, TIA, and stroke cases are designated in Fig. 2 .

## Discussion

Stroke is amongst the leading causes of fatalities and disabilities resulting from vascular defects. There is scarcity of studies aimed at elucidation of genetic variants and variances in medication intake as related to causation/risk stratification of strokes, CVAs. Current study worked on interaction of novel genetic variants, serum-based factors, and traditional vascular disease risk influences, that may ultimately manifest as stroke. We undertook tetra-primer ARMS-PCR to resolve the allelic differences, ELISA for homocysteinemia estimation, traditional kits for detecting the blood factors, and we did formal analysis through ANOVA and linear regression for detection of robust traits that associate with stroke. Hyperhomocysteinemia, differences in diastolic blood pressure readings, and MTHFR SNP rs1801133 were the main contenders that strongly related with risk of stroke/CVAs in our study.

Stroke is a debilitating disease that results in severe disability and poses a very high social and economic burden. 3 22 The traditional risk predictors associated with stroke include dyslipidemia, old age, male gender, smoking status, obesity/high fat diet, and arterial calcification of vessels supplying the central nervous system. 9 10 23 Hypercholesterolemia and hypertriglyceridemia are associated with vascular diseases and elevated stroke incidence, whereas HDL and LDL have displayed limited association with vascular diseases and resultant cerebral diseases outcomes. 10 24 In the current study, although initial analysis demonstrated that cholesterol levels had association with stroke ( *p* = 0.04) and there was significant difference in controls related to CVA cases ( *p* = 0.035), yet the regression analysis failed to maintain this significance. Serum triglyceride level was also not related to disease risk, yet HDL related to stroke risk following regression analysis. Renal disease and higher value of blood creatinine are additional influences on vascular comorbidities, are stroke predictors, and may result in fatal outcomes 25 26 ; however, creatinine did not maintain significance to stroke in present study.

Individuals with high blood pressure take various categories of medicines to limit their elevated blood pressure, to decrease risk of atherosclerosis, calcification, and to limit the additional cardiac load. There are several antihypertensive and cardiovascular risk lowering agents available, and their prescription is dependent on individual physician/patient interactions and preferences. The drugs available include lipid lowering agents (statins), blood thinners (aspirin), β receptor antagonists, calcium channel blockers, angiotensin receptor blockers/inhibitors (ARB/ARI)/ACE inhibitors, and diuretics, to name a few. The use of statins and aspirin, although not strictly antihypertensive agents, are related to depressed risk of postoperative and hypertension-mediated CVAs. 10 19 27 The use of β antagonists, calcium channel antagonists, and ACE inhibitors/ARI carry disparate results, with some studies displaying benefit and others no benefit (or high risk) in stroke prevention or adverse outcomes. 16 17 28 29 30 31 The diuretics are the mainstay of treatment in heart failure and are used as prophylaxis management regimen for stroke. An added list of literature cites use of diuretics in hypertensive patients and favorable outcomes regarding cardiac and cerebral events. 29 30 31 We studied the drug usage of all the mentioned classes of drugs and results indicate no differential drug regimen amongst the controls, TIA, and cerebral vascular disease patients.

Hypertension is harbinger of vascular diseases and stroke, with elevated readings resulting in serious complications. Elevated systolic blood pressure and elevated diastolic blood pressure readings are associated with vascular disease and various cardiovascular-related end points. 32 There are differences in outcomes and adverse effects depending on elevated levels of either of the two, SBP or DBP. 33 Studies demonstrate limited risk of heart failure, CAD, stroke, and fatal outcomes, with different readings for systolic blood pressure, and likewise different readings of diastolic blood pressure. 34 Previous studies, therefore, provide independent significance to either of the two blood pressure readings, whether SBP or DBP. The diastolic blood pressure reading was a significant factor for stroke risk in current study, as it showed significance with stroke after ANOVA ( *p* = 0.025) and also significance (through multiple comparison) in control as compared with cases, yet the SBP was nonsignificant. The regression analysis displayed limited odds when comparing relation of stroke with SBP but significant odds of stroke as a result of DBP levels, and this observation is consistent with previous studies. Homocysteine, a novel risk factor is strongly associated with CAD and stroke, with elevated levels of stroke associated with stroke risk in young. 35 Hyperhomocysteinemia was associated with stroke in current study as well ( *p* = 0.008) with significant difference when controls were compared with TIA, or with CVA cases. The odds of stroke were highly significant in hypertensive patients, following regression analysis. ( *p* = 0.019, OR = 3.075). The SNPs in folate pathway genes, rs1801133 and rs1801131 of the *MTHFR* gene, rs1805087 in MS, rs662 of PON1, and ACE gene (not in folate pathway) rs4646994 SNP have dissimilar association with stroke, as per literature. 11 12 13 14 The most extensively studied SNP in relation to stroke: *MTHFR* gene rs1801133 SNP is decisively associated with hyperhomocysteinemia, and is an additional risk enhancer for stroke. 36 None of the studied SNP, except for the rs1801133 of *MTHFR* (following regression analysis) was associated with stroke incidence in the current study. The shared minor influences of different allelic variants, medication effects, and biochemical traits may underlie the collaborative role in cerebral vascular disease consequence.

## Conclusion, Limitations, and Recommendations of the Study

Stroke is highly prevalent in Pakistan, yet the studies on elucidation of the risk factors, including novel genetic predictors or their combination effect on traditional assigned risk factors are sporadic. The current study aimed to investigate factors such as cholesterol, HDL, creatinine, and the impact of homocysteine or folate pathway gene polymorphisms with special relation to stroke in our local population.

The study comprises a restricted number of participants (particularly in the TIA group) and covers control participants who are not actually disease free, the above two being predominant constraint of the current study. The findings can be validated by an enhanced participant size, recruitment of disease-free controls, and we can address additional diversity by ensuring patient recruitment from tertiary care hospitals in more distant zones of the country. An additional improvement strategy is to study the effect of an individual drugs, and to increase the number of analyzed genes/SNPs to delineate the ones more robustly related to stroke and disease risk.
